# *XBP1* splicing triggers *miR-150* transfer from smooth muscle cells to endothelial cells via extracellular vesicles

**DOI:** 10.1038/srep28627

**Published:** 2016-06-24

**Authors:** Yue Zhao, Yi Li, Peiyi Luo, Yingtang Gao, Junyao Yang, Ka-Hou Lao, Gang Wang, Gillian Cockerill, Yanhua Hu, Qingbo Xu, Tong Li, Lingfang Zeng

**Affiliations:** 1 Department of Heart Centre, Tianjin Third Central Hospital, Tianjin 300170, China; 2 Cardiovascular Division, King’s College London BHF centre, London SE5 9NU, United Kingdom; 3 Key Laboratory of Artificial Cell, Tianjin Third Central Hospital, Tianjin 300170, China; 4 Department of Emergency Medicine, the Second Affiliated Hospital, School of Medicine, Xi’an Jiaotong University, Xi’an 710004, China; 5 St George’s University of London, London, SW17 0RE, United Kingdom

## Abstract

The interaction between endothelial cells (ECs) and smooth muscle cells (SMCs) plays a critical role in the maintenance of vessel wall homeostasis. The X-box binding protein 1 (XBP1) plays an important role in EC and SMC cellular functions. However, whether XBP1 is involved in EC-SMC interaction remains unclear. In this study, *In vivo* experiments with hindlimb ischemia models revealed that *XBP1* deficiency in SMCs significantly attenuated angiogenesis in ischemic tissues, therefore retarded the foot blood perfusion recovery. *In vitro* studies indicated that either overexpression of the spliced XBP1 or treatment with platelet derived growth factor-BB up-regulated *miR-150* expression and secretion via extracellular vesicles (EVs). The XBP1 splicing-mediated up-regulation of *miR-150* might be due to increased stability. The SMC-derived EVs could trigger EC migration, which was abolished by *miR-150* knockdown in SMCs, suggesting *miR-150* is responsible for SMC-stimulated EC migration. The SMC-derived *miR-150*-containing EVs or *premiR-150* transfection increased vascular endothelial growth factor (VEGF)-A mRNA and secretion in ECs. Both inhibitors SU5416 and LY294002 attenuated EVs-induced EC migration. This study demonstrates that XBP1 splicing in SMCs can control EC migration via SMC derived EVs-mediated *miR-150* transfer and *miR-150*-driven VEGF-A/VEGFR/PI3K/Akt pathway activation, thereby modulating the maintenance of vessel wall homeostasis.

Endothelial cells (ECs) and smooth muscle cells (SMCs) are two major cellular components within the vessel wall. ECs line the lumen forming an intact mono-layered structure that provides a semi-selective barrier between blood and the underlying tissues, and modulating vessel tone via nitric oxide production. SMCs are responsible for the contractility of the vessel wall. The liaison between ECs and SMCs plays an essential role in vessel maturation under physiological conditions, vascular injury repair and disease development such as atherosclerosis^1^,^2^.

MicroRNAs (miRNAs, miRs) are a set of small non-coding RNAs that participate in multiple cellular processes through regulating gene transcription, mRNA stability and protein translation^3^. Recent studies have revealed that miRNAs can be released from cells, delivered by extracellular vesicles(EVs) and act on recipient cells in a paracrine way, modulating intercellular communications^4^,^5^,^6^,^7^,^8^. The *miR-150* family is located in human chromosome 19. *MiR-150* has been shown to participate in multiple physiological and pathological processes, including hematopoietic cell differentiation^9^,^10^ and suppresses tumour cells proliferation^11^,^12^. In cardiovascular system, *miR-150* is reported to promote endothelial progenitor cells homing^13^ and monocyte-EC interaction^14^, while it may also be involved in the pathogenesis of pulmonary arterial hypertension^15^,^16^. In our previous study, we noticed that series of microRNA levels including *miR-150* in SMCs were up-regulated during the neointimal formation after vascular injury^17^. However, the potential role of *miR-150* in EC-SMC communication remains unclear.

The X-box binding protein 1 (*XBP1*) was originally identified as a stress-inducible transcription factor essential for cell survival under stress conditions^18^,^19^,^20^. Under endoplasmic reticulum (ER) stress conditions, *XBP1* mRNA undergoes unconventional splicing through an ER-resident kinase that possesses ribonuclease activity, the inositol requiring enzyme 1 alpha (*IRE1α*)^21^. Our recent studies and reports from other groups showed that physiological stimuli like vascular endothelial cell growth factor (VEGF) could trigger *XBP1* splicing in an ER stress response independent manner^22^,^23^,^24^,^25^. In ECs, *XBP1* splicing plays diverse roles including cell proliferation^23^, autophagy response^26^ and apoptosis^27^. In SMCs, BMP-2 was reported to activate *XBP1* splicing^28^. Our study revealed that *XBP1* splicing was activated in vascular SMCs in response to vascular injury *in vivo* and platelet derived growth factor-BB (PDGF-BB) stimulation *in vitro* and that *XBP1* deficiency in SMCs attenuated neointima formation in mouse model of femoral artery injury^17^. In this study, we demonstrated that *XBP1* splicing in SMC regulated EC migration via EVs-mediated *miR-150* transfer.

## Results

### *XBP1* deficiency in SMCs attenuated the foot blood perfusion recovery in mouse hindlimb ischemia model

**Figure 1:**
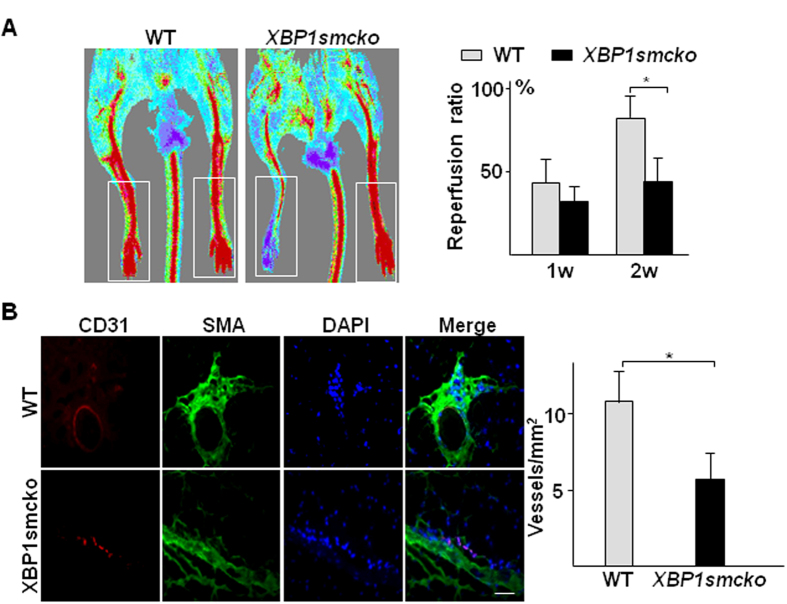
Disruption of *XBP1* gene in SMCs decreased angiogenesis in ischemic tissue. The right femoral arteries were banded at two adjacent sites with internal part cut to totally block foot blood supply in *XBP1*^*loxP/loxP*^(WT, n = 7) or SM22-*Cre*^*+*^*/XBP1*^*loxP/loxP*^(*XBP1smcko*, n = 21) mice. The blood flow (**A**) at both sides was measured by Doppler Laser Scanner at 30 minutes, 7 days and 14 days post-surgery. After blood flow measurement, the mice were humanely sacrificed and the skeletal muscle tissues around the banding site were harvested and subjected to cryo-sectioning and double immunostaining with anti-CD31 (red) and anti-SMA (green) antibodies (**B**). (**A**) Left panel shows the representative images from one mouse of each group at 14 days post-surgery. The white open box indicates the blood flow data collecting area. The right panel shows the average blood flow perfusion ratio of the right to the left foot from all mice in each group. (mean ± SEM, *P < 0.05; ANOVA, Dunnett post-test). (**B**) Left panel shows the representative images of blood vessels in the skeletal muscle tissues from one mouse of each group at 14 days post-surgery. Nucleus was conterstained with DAPI. Scale bar: 100 μm. Right panel shows the average vessel density (all vessels including capillary vessels) in the tissues collected from all mice in each group. (mean ± SEM, *P < 0.05; Student T-test).

To test whether *XBP1* was involved in EC-SMC interaction, we performed a hindlimb ischemia model in *SM22-Cre/XBP1*^*loxP/loxP*^ (*XBP1smcko*) mice and observed foot blood perfusion recovery. Compared to *XBP1*^*loxP/loxP*^ mice, the foot blood perfusion recovery in the injured limb was significantly attenuated in mice at 2 weeks post-surgery (Fig. 1A) as revealed by Doppler scanning analysis. Immunofluorescence staining with anti-CD31 and anti-αSMA antibodies revealed that the capillary vessel density was significantly decreased in the injured skeletal muscle in *XBP1smcko* mice as compared to *XBP1*^*loxP/loxP*^ mice (Fig. 1B). These results suggest *XBP1* deficiency in SMCs may affect angiogenesis, therefore causing the retardation of the foot blood perfusion recovery.

### Overexpression of *XBP1s* in SMCs triggered EC migration via paracrine mechanisms

**Figure 2:**
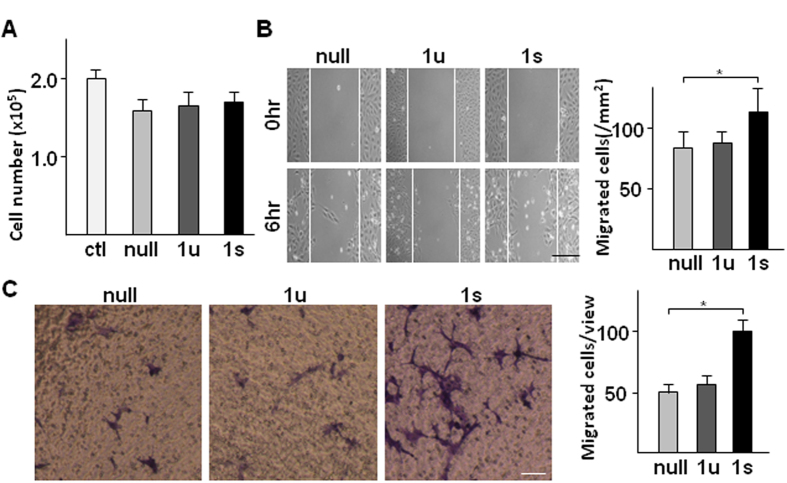
*XBP1* splicing in SMCs increased EC migration via secreted factors. HSMCs were infected with Ad-null (null), Ad-*XBP1s* (1s), or Ad-*XBP1*u (1u) at 10 MOI for 24 hr. The medium was then replaced by M199 medium supplemented with 0.5% FBS and incubated for 8 hr. The conditioned medium was then applied to HUVECs for proliferation assays via cell number counting (**A**) 24 hr post incubation) and cell migration assessments via wound healing assays (**B**) 6 hr post incubation, scale bar: 200 μm) and transwell migration assays (**C**) 6 hr post incubation, scale bar: 25 μm). Data presented are representative images or mean ± SEM of three independent experiments. (*P < 0.05; ANOVA, Dunnett post-test).

During the angiogenesis process, the initial step is the migration of ECs out of the intact endothelium, followed by EC proliferation. As described above, *XBP1* deficiency in SMC affected angiogenesis in ischemic tissue, indicating that *XBP1* in SMC may be involved in EC migration and proliferation via a paracrine mechanism. To test this, the conditioned medium was collected from SMCs infected with Ad-*XBP1u* and Ad-*XBP1s* viruses respectively, and applied to HUVECs to assess cell proliferation and migration. Cell number calculation assays showed that there was no difference among the conditioned media from SMCs infected with either of the two viruses or the control virus (Ad-null) (Fig. 2A), suggesting that *XBP1* in SMC has no effect on EC proliferation. There was a decrease of cell proliferation under conditioned media as compared to fresh medium, which might be due to prior nutrient consumption by SMCs in the conditioned media. However, the EC monolayer wound healing assay revealed that the conditioned medium from SMCs infected with Ad-*XBP1s*, but not those from cells infected with Ad-*XBP1*u-infected, had significantly increased EC migration (Fig. 2B). This was further confirmed by transwell-based migration assay (Fig. 2C), suggesting that *XBP1* splicing in SMCs may regulate EC migration through some secreted factors.

### *MiR-150* was mediated by overexpression of *XBP1s* in SMCs-induced EC migration

**Figure 3:**
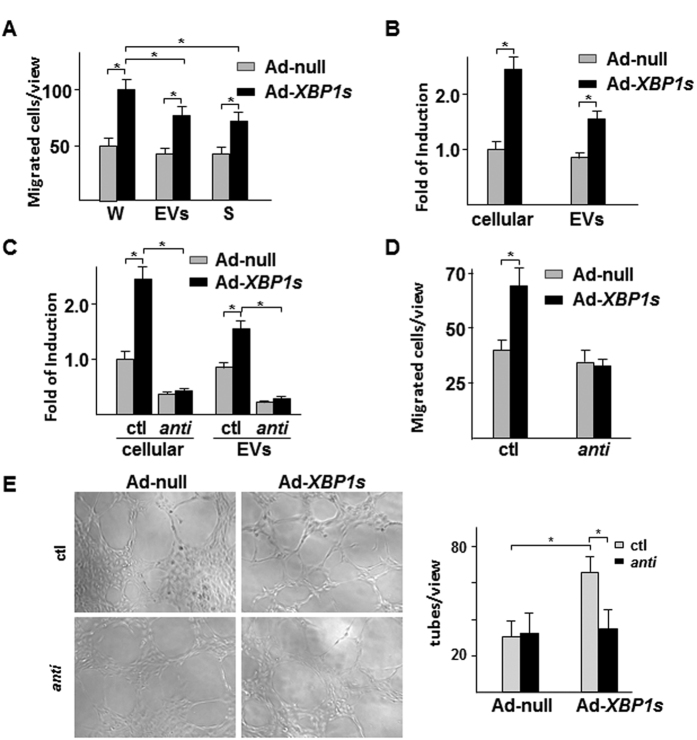
miR*-150* was mediated by *XBP1* splicing in SMC-induced EC migration. (**A**) Effect of the whole conditioned medium (W), EVs and supernatant (S) on HUVEC migration in transwell assay. (**B**) Over-expression of *XBP1s* in SMC increased *miR-150* expression and secretion in EVs. (**C**) *anti-miR-150* abolished *XBP1s*-induced *miR-150* expression and secretion. (**D**) *anti-miR-150* abolished *XBP1s*/SMC-derived EVs-induced HUVEC migration. Data presented are mean ± SEM of three independent experiments. (**E**) *anti-miR-150* abolished *XBP1s*/SMC-derived EVs-induced HUVEC tube formation. Left panel shows the representative images. Right panel shows the average tubes/10x view from 3 views/well of 3 wells (*P < 0.05; ANOVA, Dunnett post-test).

To analyse the potential responsible factors in the conditioned medium, EVs was isolated by ultracentrifugation. The effect of the whole conditioned medium, EVs and EVs-depleted medium on EC migration was performed with the transwell migration assay. As shown in Fig. 3A, both EVs and EVs-depleted medium from Ad-*XBP1s*-infected SMCs significantly increased EC migration, suggesting that stimulating factors exist in both EVs and medium. In our previous study, we found that activation of *XBP1* splicing modulated multiple microRNA levels in SMCs, of which *miR-150* was up-regulated^17^. It has been reported that *miR-150* was involved in monocyte-regulated EC migration^14^,^29^. Therefore, we wondered whether *miR-150* contributed to EVs-mediated pro-migratory effect. Quantitative RT-PCR analysis confirmed that overexpression of *XBP1s* in SMCs increased cellular and EVs *miR-150* levels (Fig. 3B). Transfection of SMCs with *anti-miR-150* oligos abolished overexpression of *XBP1s*-mediated upregulation of *miR-150* in both whole cell lysates and EVs (Fig. 3C). As expected, the *anti-miR-150* transfection in SMCs abolished the EC pro-migratory effect of the EVs isolated from Ad-*XBP1s*-infected SMCs as compared to control anti-mir RNA oligos (Fig. 3D). Tube formation is another criterion to evaluate EC functions in angiogenesis. Our studies revealed that EVs isolated from Ad-*XBP1s*-infected HSMCs significantly increased HUVEC capillary structure formation on Matrigel, which was ablated by *anti-miR-150* transfection in SMCs (Fig. 3E). These results suggest that *miR-150* plays an essential role in EVs-mediated EC migration and angiogenesis.

### PDGF upregulated *miR-150* in SMCs in *XBP1* splicing dependent manner

**Figure 4:**
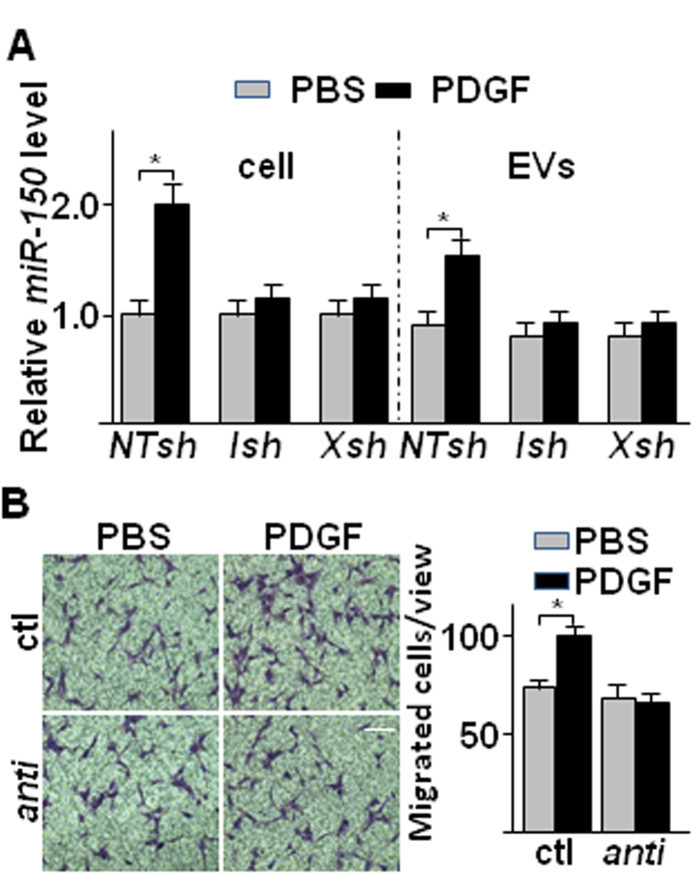
PDGF up-regulated *miR-150* expression and secretion in SMC in *IRE1α*/*XBP1* dependent manner. (**A**) PDGF increased *miR-150* expression and secretion in SMC in an *XBP1* splicing dependent manner. HSMCs were infected with non-target (*NTsh*), *IRE1α* (*Ish*) or *XBP1* (*Xsh*) shRNA lentivirus at 100 IU for 48 hr, and then treated with DMEM supplemented with 0.5% FBS for 24 hr, followed by 20 ng/ml PDGF-BB treatment for 4 hr. The cellular and EV *miR-150* levels were assessed by quantitative RT-PCR of three independent experiments. (mean ± SEM, *P < 0.05; ANOVA, Dunnett post-test) (**B**) The *anti-miR-150* abolished PDGF/SMC-mediated EC migration. HSMCs was transfected with *anti-miR-150* RNA (*anti*) and incubated in complete growth medium for 48 hrs, and then treated with 0.5% FBS medium for 24 hr, followed by 20 ng/ml PDGF-BB treatment for 4 hr. The effect of isolated EVs on HUVEC migration was assessed by transwell migration assays. The left panel shows the representative images, while the right panel shows the statistical analysis of migrated cells per 10x view. Control *anti-miR* RNA (ctl) was included. Scale bar: 25 μm. Data presented are representative images or mean ± SEM of three independent experiments. (*P < 0.05; ANOVA, Dunnett post-test).

To test whether *miR-150* could be upregulated in SMCs under physiological or pathological conditions, the effect of PDGF has been examined. Our previous studies have revealed that PDGF treatment triggered *XBP1* splicing via *IRE1α* phosphorylation^17^. Hence SMCs were infected with *IRE1α* or *XBP1* shRNA lentivirus and treated with PDGF, followed by analysis of *miR-150* in whole cell lysate and EVs. As shown in Fig. 4A, PDGF treatment increased *miR-150* in both the cell lysate and EVs from non-target shRNA (*NTsh*) infected SMCs. However, the increase was abolished by either *IRE1α* shRNA (*IRE1αsh)* or *XBP1* shRNA (*XBP1sh*). These results suggest that *IRE1α*-mediated *XBP1* splicing is essential for PDGF-induced *miR-150* upregulation and secretion via EVs. The EVs isolated from PDGF-treated SMCs increased EC migration, which was abolished by *anti-miR-150* transfection (Fig. 4B), suggesting that *miR-150* is involved in PDGF-mediated EC/SMC interaction.

### *XBP1s* increased *miR-150* stabilization

**Figure 5:**
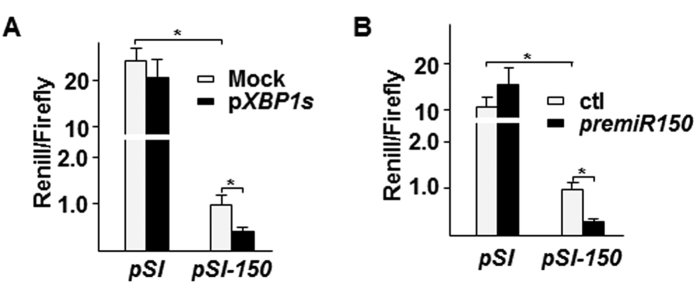
*XBP1s* suppressed *miR-150* transcription. (**A**) Over-expression of *XBP1s* suppressed *miR-150* promoter reporter gene expression. 0.1 μg/well pSi-CHECK2-*miR-150* (*pSI-150*) was co-transfected with 0.1 μg/well pShuttle2-*XBP1s* (p*XBP1s*) into HSMCs in triplicate. Renilla and Firefly luciferase activities were assessed 48 hr post-transfection. pSi-CHECK2 (*pSI*) and pShuttle2 (Mock) were included as control. (mean ± SEM, *P < 0.05; ANOVA, Dunnett post-test) (**B**) Exogenous *premiR-150* suppressed *miR-150* promoter reporter gene expression. 0.1 μg/well pSi-CHECK2-*miR-150* (*pSI-150*) was co-transfected with 10 pmol/well *premiR-150* into HSMCs in triplicate. Renilla and Firefly luciferase activities were assessed 48 hr post-transfection. pSi-CHECK2 (*pSI*) and *premiR* control (ctl) were included as control. Data presented are mean of three independent experiments. (mean ± SEM, *P < 0.05; ANOVA, Dunnett post-test).

*XBP1* is a zinc leucine zip transcription factor. Our previous study has shown that *XBP1* splicing in SMCs increased mir-1274B via direct transcriptional regulation^17^. We wondered whether a similar mechanism exists in *miR-150* upregulation. To test this, we cloned a 1.1 kb fragment corresponding to the promoter region of the *miR-150* gene into pSI-Check2 vector to replace the SV40 early enhancer promoter. Co-transfection of this plasmid with *XBP1s* overexpressing plasmid (pShuttle2-*XBP1s* (p*XBP1s*)) was performed in SMCs, followed by luciferase activity assays. Surprisingly, overexpression of *XBP1s* did not increase the reporter gene expression, but instead decreased the reporter gene expression (Fig. 5A). Chromatin-immunoprecipitation assay did not show a direct binding of *XBP1s* to the promoter region(data not shown). Therefore, we assumed that *XBP1s* increased *miR-150* stability, leading to feedback inhibitory effect on its transcription. Indeed, when the reporter plasmid was co-transfected with *miR-150* pre-mir mimic, the reporter gene expression was significantly decreased (Fig. 5B), suggesting a feedback inhibitory effect exists.

### *MiR-150* activated VEGF-A autocrine/paracrine-mediated VEGF receptor/PI3K-Akt signalling pathway

**Figure 6:**
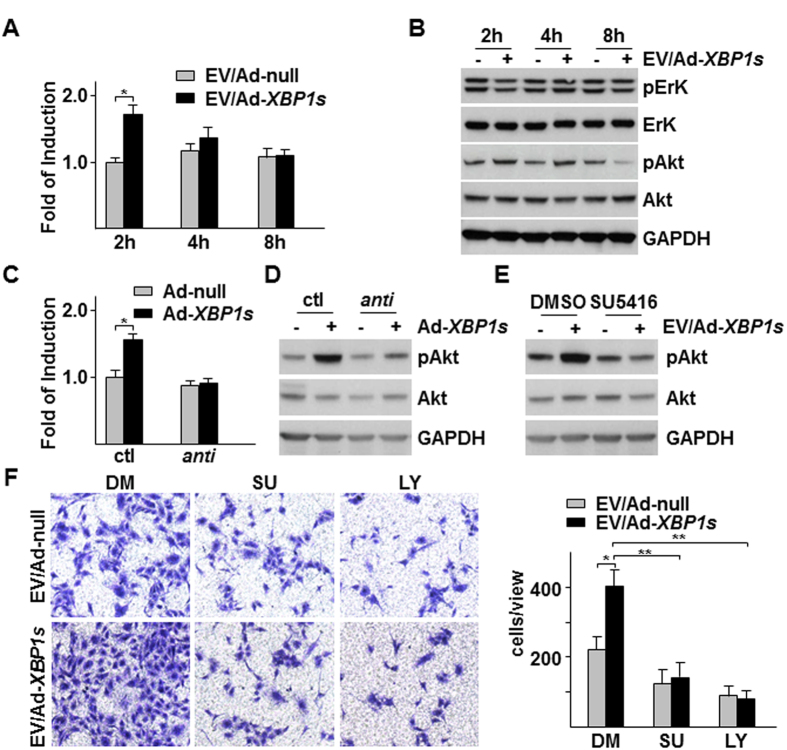
VEGF-PI3K/Akt pathway was responsible for Ad-*XBP1s*/HSMC-derived EVs-mediated EC migration. (**A,B**) Ad-*XBP1s*/HSMC-derived EVs increased *VEGF-A* mRNA and Akt phosphorylation in HUVECs. EVs were isolated from Ad-*XBP1s*/HSMC condition medium (CM), reconstituted in M199 medium supplemented with 5 μg/ml insulin and 5 μg/ml transferrin and applied to HUVECs for time indicated, followed by quantitative RT-PCR analysis of *VEGF-A* mRNA (A) or western blot analysis of ErK and Akt (S473) phosphorylation (**B**). (**C,D**) *Anti-miR-150* abolished *XBP1s*/HSMC-derived EVs-mediated VEGF-A expression and Akt. EVs were isolated from control (ctl) or *anti-miR-150* transfected with Ad-*XBP1s*-infected HSMCs CM, reconstituted and applied to HUVECs for 2 h, followed by *VEGF-A* mRNA (**C**) and Akt (S473) phosphorylation assays (**D**). (**E**) SU5416 ablated Ad-*XBP1s*/HSMC-derived EVs-induced Akt phosphorylation. HUVECs were treated with EVs isolated from Ad-*XBP1s* in the presence of 10 μM SU5416 for 2 h, followed by Akt phosphorylation assay. (**F**) SU5416 and LY294002 attenuated Ad-*XBP1s*/HSMC-derived EVs-induced HUVEC migration. Transwell migration assays were performed with EVs isolated from Ad-*XBP1s*-infected HSMC CM in M199 medium supplemented with insulin and transferrin in the presence of SU5416 (SU) or 10 10 μM LY294002 (LY) for 8 h. Left panel shows the representative images of migrated cells while right panel shows average migrated cells per 10x view. Ad-null and DMSO (DM) were included as control. Data presented are representative images or mean of three independent experiments. (mean ± SEM, *P < 0.05, **P < 0.01; ANOVA, Dunnett post-test).

It was reported that microvesicle-derived *miR-150* could promote tumorigenesis via up-regulating VEGF^30^. VEGF is a well-known EC activator, modulating multiple EC functions including migration. Thus, we wondered whether there was a crosstalk between *miR-150* and VEGF in our system. We firstly assessed the effect of Ad-*XBP1s*/HSMC-derived EVs on the VEGF-A mRNA in HUVECs and found that the EVs transiently upregulated VEGF-A mRNA level (Fig. 6A). Western blot analysis revealed that Ad-*XBP1s*/HSMC-derived EVs exerted different effect on two VEGF/VEGF receptor downstream signal pathways, ErK and Akt pathways. The EVs increased Akt phosphorylation but decreased ErK phosphorylation (Fig. 6B). Knockdown of *miR-150* by *anti-miR-150* transfection in HSMCs abolished *XBP1s*/HSMC-derived EVs-induced *VEGF-A* mRNA upregulation (Fig. 6C) and Akt phosphorylation (Fig. 6D), suggesting that EVs-mediated *VEGF-A* upregulation and Akt phosphorylation is *miR-150* dependent. VEGF receptor inhibitor SU5416 abolished EV-induced Akt phosphorylation in HUVECs (Fig. 6E), suggesting that VEGF-A may function as an autocrine or paracrine factor for Akt activation. Further experiments demonstrated that SU5416 and PI3K inhibitor LY294002 significantly decreased Ad-*XBP1s*/HSMC-derived EVs-induced EC migration as revealed by transwell migration assays (Fig. 6F). These results suggest that *miR-150*-mediated VEGF-A upregulation and VEGFR/Akt activation are responsible for EV-induced EC migration.

**Figure 7:**
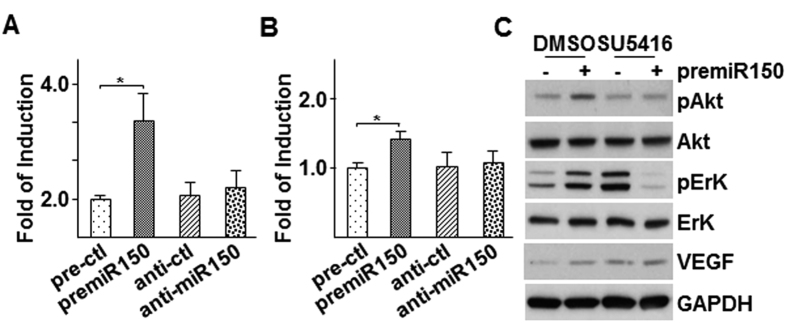
Over-expression of *premiR-150* increased VEGF-A expression and VEGF receptor dependent Akt phosphorylation in HUVECs. (**A,B**) Over-expression of *premiR-150* increased VEGF-A expression and secretion. HUVECs were transfected with *premiR-150* or *anti-miR-150* in serum free M199 medium for 5 h, then treated with M199 medium supplemented with 5 μg/ml insulin and 5 μg/ml transferrin for 3 h, followed by quantitative RT-PCR analysis of VEGF-A mRNA (**A**) or ELISA assay to detect VEGF-A in cell culture medium (**B**) *premiR* and *anti-miR* control RNA fragments were included as controls. (**C**) SU5416 ablated *premiR-150*-induced Akt and ErK phosphorylation. HUVECs were transfected with *premiR-150* or *anti-miR-150* in serum free M199 medium for 5 h, then treated with M199 medium supplemented with 5 μg/ml insulin and 5 μg/ml transferrin in the presence of 10 μM SU5416 for 3 h, followed by Western blot analysis of Akt and ErK phosphorylation and VEGF expression. premiR RNA fragments DMSO were included as controls. Data presented are representative images or mean of three independent experiments. (mean ± SEM, *P < 0.05; ANOVA, Dunnett post-test).

To further confirm the involvement of *miR-150* in VEGF-A mRNA regulation and Akt activation in ECs, we transfected HUVECs directly with *premiR-150* or *anti-miR-150* followed by VEGF-A mRNA, VEGF-A secretion in medium and VEGF-A expression and Akt action in the cells within 3 hr post transfection. As shown in Fig. 7A, *premiR-150* transfection significantly increased VEGF-A mRNA while *anti-miR-150* transfection had no effect. ELISA analysis of VEGF-A in cell culture medium revealed a similar trend (Fig. 7B). Western blot analysis indicated that *premiR-150* transfection increased both ErK and Akt phosphorylation, which was ablated by the presence of SU5416 (Fig. 7C). A significant increase was detected in VEGF band in the *premiR-150* transfection sample, while elevated amount of VEGF could be detected in both control and *premiR-150* transfection samples under SU5416 treatment (Fig. 7C). The results suggest that *miR-150* can upregulate VEGF-A mRNA, secretion and its receptor-mediated Akt phosphorylation in ECs.

## Discussion

The communication between ECs and SMCs is critical for the maintenance of vessel wall homeostasis and remodelling. Under physiological conditions, ECs and SMCs liaise with each other via paracrine and/or juxtacrine manner to control vessel tone^31^. The co-ordinations between ECs and SMCs on their proliferation and migration are essential for arteriogenesis^32^. Under pathological conditions, risk factors-induced endothelial dysfunction can trigger medial SMC proliferation and/or apoptosis, eventually leading to vascular disease development^33^,^34^. Multiple genes and factors have been found to be involved in the liaison of EC-SMC interaction. *XBP1* plays diverse roles in ECs and SMCs. In ECs, under oxidative or ER stress, *XBP1* splicing contributes to EC autophagic and apoptosis response^26^,^27^,^35^,^36^,^37^; while under growth factor stimulation, *XBP1* splicing contributes to EC proliferation^23^,^24^,^38^. In SMCs, *XBP1s* contributes to calponin h1 decrease, leading to SMC proliferation and neointima formation^17^,^39^. In this study, we demonstrate that *XBP1* is also involved in the liaison between ECs and SMCs.

The compensative angiogenesis in ischemic tissue is essential for cell survival and tissue or organ function restoration. This is a multiple cells and factors-involved multi-step process, including existing ECs-mediated angiogenesis and stem/progenitor cell-mediated neovascularization. VEGF, PDGF and hypoxia inducible factor 1 (HIF-1) are the main modulators of angiogenesis.

Several studies have shown that *XBP1* promotes angiogenesis in cardiac hypertrophy via upregulating VEGF expression in ECs^25^,^40^,^41^,^42^,^43^. Our previous study revealed that *XBP1* is a downstream of VEGF signalling pathway mediator^23^. In this study, we found that *XBP1* splicing in SMCs could upregulate VEGF-A expression and VEGF receptor-dependent Akt phosphorylation in ECs. The activation of Akt phosphorylation may be modulated through VEGF via autocrine or paracrine mechanisms. The overall effect is to induce EC migration and angiogenesis. The key mediator for the *XBP1*/VEGF crosstalk-mediated EC-SMC liaison seems to be *miR-150.* MiRNAs are a set of small non-coding RNAs participating in multiple cellular processes including cell motility. Secretion and delivery of miRNAs via EVs contribute to intercellular communication^44^. It has been reported that *miR-150* can be secreted from monocytes via EVs and increase EC migration and angiogenesis^14^,^29^,^30^. Our study demonstrated that *miR-150* can be secreted from SMCs via EVs and increase VEGF-A expression and Akt phosphorylation in ECs. Very importantly, direct transfection of *miR-150* into HUVECs can increase VEGF-A mRNA, VEGF secretion and Akt phosphorylation. These results suggest a positive role of *miR-150* in VEGF-A signalling pathways and EC migration and angiogenesis. Different from *miR-150* transfection-induced ErK phosphorylation, SMC-derived EVs suppressed ErK phosphorylation in EC, which may be due to other inhibitory components within the EVs. This may also explain why EVs have no effect on EC proliferation. However, several reports have suggested that endothelial intrinsic *miR-150* seems to play a negative role in EC migration and/or angiogenesis^45^,^46^,^47^. The discrepancy among these reports may be derived from different protocols. In our study, we assessed VEGF-A expression, secretion and VEGF-downstream signalling pathway activation within 3 hours post transfection. A negative feedback may exist in a long term period.

PDGF is another important growth factor for angiogenesis under ischemia. It can be induced from ECs in response to ischemia or other stimulus^48^,^49^,^50^, which in turn activates medial SMCs proliferation and migration, leading to vessel wall remodelling^51^,^52^. During this process, *XBP1* splicing plays an important role^17^. In the present study, we found that PDGF stimulated SMCs to secret *miR-150*-containing EVs in *XBP1* splicing dependent manner, leading to EC migration. Therefore, we may speculate that PDGF functions as a liaison mediator from ECs to SMCs.

HIF-1 is a critical transcription factor, essential for cell survival and angiogenesis under ischemia. In response to hypoxia under ischemia conditions, HIF-1 upregulates the transcription of a set of genes that are involved in cell proliferation in both ECs and SMCs, contributing to revascularization in the ischemic tissues^53^. It has been reported that *XBP1* can form a complex with HIF-1 in regulating gene transcription in cancer cells^54^. HIF-1 was reported to negatively regulate *miR-150* expression during liver regeneration. In this study, we found that *XBP1* suppressed *miR-150* transcription, in which HIF-1 might be involved. *XBP1*/HIF-1 complex may be responsible for the *miR-150* downregulation in pulmonary airway SMCs under hypertension^16^. Further detailed investigation will be required to address the crosstalk between *XBP1* and HIF-1 in both ECs and SMCs following ischemia.

**Figure 8:**
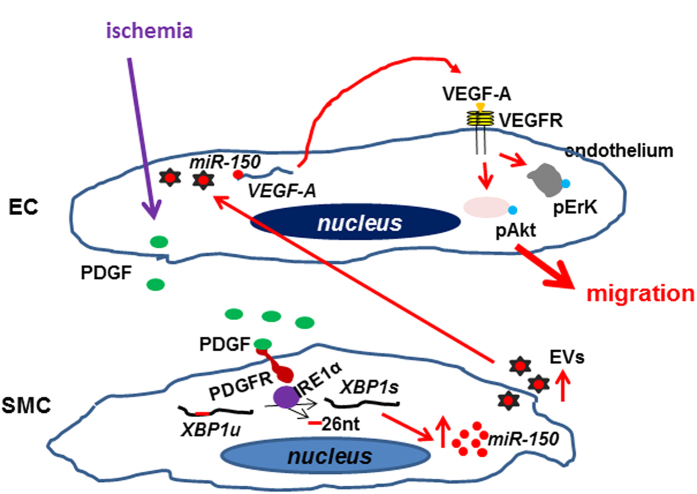
A schematic illustration of PDGF-*IRE1α*-*XBP1*-*miR-150* pathway in EC-SMC interaction. Under ischemia, the activated ECs may secret PDGF, which in turn activates *IRE1α* phosphorylation, leading to *XBP1* mRNA splicing to remove 26-nucleotide intron in SMCs. The spliced *XBP1* stabilizes *miR-150* and increases its secretion via extracellular vesicles (EVs). The *miR-150*-containing EVs are taken by ECs, increasing VEGF-A mRNA transcription or stability, leading to VEGF-A protein production and secretion. VEGF-A triggers the ErK and Akt phosphorylation via binding to VEGF receptor in an autocrine or paracrine manner. The activated Akt contributes to EC migration, leading to angiogenesis.

Taken together, we may speculate that under ischemia the activated ECs secrete PDGF, which in turn activates *XBP1* splicing in SMCs. The spliced *XBP1* stabilizes *miR-150* and increases *miR-150* secretion via EVs. The *miR-150*-containing EVs are taken by ECs. The *miR-150* upregulates VEGF-A mRNA and protein expression and secretion. VEGF activates Akt phosphorylation via an autocrine or paracrine mechanism, leading to ECs migration and angiogenesis (Fig. 8). Thus, PDGF, *XBP1* splicing, *miR-150* and VEGF form an important signaling pathway to liaise EC-SMC interaction, in which *XBP1* splicing plays a central role and HIF-1 may be also involved through interaction with *XBP1*.

So far, we have demonstrated that *XBP1* splicing is involved in EC and SMC proliferation and their interaction, similar to many other important molecules, like PDGF, HIF-1. This will require us to make critical thinking when we intend to develop therapeutic strategies to intervene human diseases. For example, if we want to target *XBP1* splicing in SMCs to suppress airway SMC proliferation under pulmonary hypertension, it may affect the EC-SMC interaction and EC proliferation as well. So, a cell specific targeting strategy will be required.

## Methods

### Chemicals and Reagents

All cell culture serum and media were purchased from Thermo Fisher Scientific, while cell culture supplements were purchased from Sigma. Rat anti-CD31 (553369) was purchased from Pharmingen while antibodies against alpha SMA (ab7817), phosphor-ErK (ab50011) and ErK1/2 (ab17942) were from Abcam. The antibodies against phospho-Akt (sc-7985R), Akt1 (sc-1619) and GAPDH (sc-25778) were from Santa CruZ Biotech. The antibody against VEGF (SAB1402390) was from Sigma. All secondary antibodies were from Dakocytomation. All microRNA reagents were purchased from Thermo Fisher Scientific. PDGF, insulin, holo-transferrin, SU5416, LY294002, DMSO, DAPI and Giemsa were purchased from Sigma.

### Cell culture

Human aortic smooth muscle cells (HSMC, ATCC-PCS-100-012) were purchased from ATCC and cultured on 0.04% gelatin-coated flasks in DMEM medium supplemented with 10% fetal bovine serum (FBS), 100 U/ml penicillin and streptomycin in humidified incubator supplemented with 5% CO_2_. The cells were split every three days at a ratio of 1:3. Human umbilical vein ECs (HUVECs, ATCC-PCS-100-010) were cultured on 0.04% gelatin-coated flasks in M199 medium supplemented with 1 ng/ml β-EC growth factor, 3 μg/ml EC Growth Supplement from bovine neural tissue, 10 μ/ml heparin, 1.25 μg/ml thymidine, 10% FBS, 100 μ/ml penicillin and streptomycin in humidified incubator supplemented with 5% CO_2_. The cells were split every three days at a ratio of 1:5. Both HSMCs and HUVECs up to passage 10 were used in this study. HEK293 and HEK293T cells were maintained in DMEM supplemented with 10% FBS and penicillin/streptomycin and were split every three days at a ratio of 1:4.

### Adenoviral and shRNA lentiviral infection

For adenoviral infection, HSMCs were incubated with Ad-null, or Ad-*XBP1u*(unspliced *XBP1*) *or* Ad*-XBP1s*(spliced *XBP1*) virus at 10 MOI for 6 hr, and then cultured in fresh complete growth medium for time duration indicated in figure legends. For shRNA lentiviral infection, HSMCs were incubated with 100 transfection unit/cell of non-target shRNA or *XBP1* shRNA or *IRE1α* shRNA lentivirus in the presence of 10 mg/mL polybrene for 16 hr, followed by culture in fresh complete growth medium for 72 hr and subjected to further treatments.

### Cell counting

HUVECs were seeded in complete growth medium at a 1 × 10^5^ cells/well in 6-well plates for 24 hr. The medium was changed to M199 supplemented with 0.5% FBS (control)-based conditioned medium from the Ad-*XBP1s* infected SMCs and incubated for 24 hr. Upon harvest, the cells were trypsinized to single cell suspension and subjected to cell counting using a multisizer 3 coulter counter (Beckman Coulter), according to the manufacturer’s instructions.

### Wound healing migration assay

Wound was created by scratching confluent HUVECs in 6-well plates. After removal of the cell debris and medium, fresh conditioned medium was added and incubated for 6 hr. Three scratching lines were created in each well, and each conditioned medium was performed in three wells. Images were taken with at three different sites on each scratching line at 0 hr and 6 hr. The average migrated cells were calculated from 3 sites/line x 3 lines/well x3 wells.

### Transwell migration assay

The HUVECs were detached by using trypsin and resuspended in M199 containing 0.5% FBS. A 100 μl of 5 × 10^5^ cells/ml cell suspension was added into the insert and 600 μl of M199 containing 0.5% FBS or conditioned medium or reconstituted EVs was added into the holder of the transwell (8 μm pore size), followed by incubation for 6 hr. The cells were then fixed with methanol/acetic acid (3:1) for 15 minutes, and stained with Giemsa solution (Sigma) for 15 minutes. After removal of the cells inside the insert, the migrated cells were observed under Nikon Eclipse TS100 microscope and images were taken by Nikon Digital Sight system and processed with Adobe Photoshop software. Cells were calculated from 3 10x view/well x 3 wells.

### Tube formation assay

The effect of EVs on EC angiogenesis was assessed by tube formation assay as described previously^55^. Briefly, 50 μl/well of growth factor-reduced Matrigel solution (Millipore) was added to 96-well plate and solidified at 37 °C. EVs were isolated from *anti-miR-150* or control miR-transfected Ad-null or Ad-*XBP1s*-infected HSMCs and reconstituted in M199 medium supplemented with 5 μg/ml insulin (Sigma) and 5 μg/ml transferrin (Sigma). 3000 HUVECs were resuspended in 100 ul EV-reconstituted medium and added to matrigel containing wells in triplicate and incubated in 37 °C humidified incubator supplemented with 5% CO_2_ for 4 h. Tube formation was observed under Nikon Eclipse TS100 microscope and images were taken by Nikon Digital Sight system and processed with Adobe Photoshop software. Cells were calculated from 3 10x view/well x 3 wells.

### EVs isolation

The EVs were isolated from SMCs culture supernatants by differential centrifugations as previously described^56^. Briefly, the conditioned medium was collected from Ad-*XBP1s*-infected or PDGF-BB treated SMCs and centrifuged at 500 g (Sorvall Legend RT,75006445 swing-out rotor) at 4 °C for 5 min to remove cell debris. The supernatant was collected and centrifuged again at 4500 g at 4 °C for 30 min to remove smaller debris. The supernatant was then transferred to Ultra-Clear™ tube (Beckman coulter, Item No: 344059) and ultracentrifuged at 120000 g (Hitachi CP100NX,TH-641 rotor) at 4 °C for 60 min. Pellets were resuspended and washed in 1 ml of cold PBS and ultracentrifuged again at the same condition. The EVs pellets were subjected to miRNAs isolation and quantitative RT-PCR analysis for *miR-150* or reconstituted into the original volume with M199 medium containing 0.5% FBS (Figs 2, 3, 4 except Fig. 3E) or M199 medium supplemented with 5 μg/ml insulin (Sigma) and 5 μg/ml transferrin (Sigma) (Figs 3E, 6 and 7) for cellular function assays.

### MicroRNA analysis

The cellular total RNA was isolated with mirVana™ miRNA isolation kit according to the manufacturer’s instruction. The reverse transcription of miRNAs was performed with the Applied Biosystems^®^ TaqMan^®^ MicroRNA Reverse Transcription kit and Applied Biosystems^®^ 5x RT primer. The quantitative PCR amplifications of samples were done via Applied Biosystems^®^ TaqMan^®^ Universal PCR Master Mix, No AmpErase^®^ UNG and 20x TaqMan small RNA Assay with protocol provided.

### VEGF-A mRNA analysis

The cellular total RNA was isolated with Qiagen RNeasy kit with protocol provided. One microgram of RNA was reverse transcribed into cDNA using Impro-RT transcription system (Promega) and 20 ng cDNA (relative to RNA amount) was amplified by a real time PCR SYBR master mix (Applied Biosystems) with a primer set of 5′> ctg tct tgg gtg cat tgg agc <3′ and 5′> ctg cat ggt gat gtt gga ctc <3′ for VEGF-A. The 18s rRNA primer set of 5′> ata cat gcc gac ggg cgc tg <3′ and 5′> gga gag ggg ctg acc ggg tt <3′ was included as internal control. The fold of induction was defined as ct for VEGF-A minus ct for 18s rRNA with that of control group set as 1.0.

### Plasmid construction and transient transfection

The 1.1 kb fragment of *miR-150* promoter region was amplified by PCR using a primer set of 5′> gcg cag atc tat tca ctt aat taa aga caa aga g <3′ and 5′> tat atc tag agc cgc cgc tgc cgc tgc tgc ctc g <3′ and inserted into the *BglII*/*NheI* sites of pSi-CHECK2 to replace the SV40 early enhancer promoter, designated as *pSI-150*. The plasmid was verified by DNA sequencing. For transient transfection assay, HSMCs were seeded in 12-well plates at 5 × 10^4^ cells/well in triplicate 24 hr prior to transfection. Medium was changed to serum and antibiotics free DMEM. 0.1 μg/well reporter plasmid DNA (pSi-CHECK2 or *pSI-150*) and 0.1 μg/well pShuttle2-*XBP1s* or 10 pmol *premiR-150* were transfected into HSMCs with 1 μl lipofectamine RNAiMax (Thermo Fisher Scientific) according to the procedure provided. Five hours later, the transfection solution was removed and fresh complete growth medium was added and the cells were incubated for 24 hr, followed by luciferase activity assay with the dual reporter system (Promega) according to the protocols provided. The relative luciferase activity was defined as the ratio of readout for Renilla luciferase to that for Firefly luciferase with that of control group set as 1.0. For the knockdown of endogenous *miR-150*, HSMC were transfected by 50 pmol/25 ml flask *anti-miR-150* (hsa-*miR-150*-5p inhibitor, Sigma) with lipofectamine RNAiMax (Thermo Fisher Scientific) according to the procedure provided.

### ELISA analysis of VEGF

HUVECs were transfected with *premiR* control, *premiR-150*, *anit-miR* control or *anti-miR-150* in serum free M199 medium for 5 h, followed by incubation with M199 medium supplemented with 5 μg/ml of insulin and 5 μg/ml of transferrin for 3 h. The medium was collected and cell debris was removed by centrifugation at 2000 rpm at 4 °C for 5 min. The medium was then concentrated 20 fold using Ultracel-10 K centrifuge unit (Millipore) and subjected to VEGF concentration assessment using VEGF human ELISA Kit (ab100663, Abcam) with protocol provided. The fold of induction was defined as the relative A450 nm absorbance with that of *premiR* control group set as 1.0.

### Western blot analysis

Western blot analysis was performed according to standard procedures described elsewhere. Briefly, the cells were lysed with IP-A buffer [10 mM Tris-HCl, pH7.5, 120 mM NaCl, 1 mML EDTA, 1% Triton X-100 plus protease inhibitor cocktail tablets (one tablet for 50 mL, Roche)] and the protein concentration was measured by BioRad protein assay (BioRad)). 50 μg lysate was applied to SDS-PAGE, PVDF membrane (Amersham) transfer, blocking with 5% milk powder in TBST buffer (10 mM Tris-HCl, pH7.5, 120 mM NaCl, 1 mM EDTA, 0.05% Tween-20), primary and secondary antibodies incubation, ECL development and X-ray film exposure (Amersham). The images were processed by Adobe Photoshop software.

### Hindlimb ischemia

The *XBP1*^*LoxP/LoxP*^(regarded as wild type, *WT*), and *SM22Cre/XBP1*^*loxP/loxP*^ (SMC conditional knockout, *XBP1smcko*) mice were created as described previously^17^ and were anaesthetized using a combination of Hypnorm (25 mg/kg; Veta Pharma, UK) and Hypnovel (25 mg/kg; Roche). The right femoral artery was ligated permanently, i.e. the femoral artery was ligated at two adjacent sites with the middle part cut^23^. The foot blood flow was measured with LDI Doppler laser scanner (Moor Instruments) 30 minutes post-surgery, 1 week and 2 weeks post-surgery. The reperfusion percentage was defined as the ratio of mean measurement in the foot area of ligated hindlimb to that of contralateral unligated hindlimb. The data was processed with Microsoft Excel software. The flow image was processed with Adobe Photoshop software. The mice were sacrificed humanely after flow measurement at 2 weeks post-surgery. Adductor muscle tissues of ligated side were harvested and cryo-sectioned, followed by immunofluorescent staining with anti-CD31 and smooth muscle actin antibodies. All animal experiments in this study were performed according to protocols approved by the Institutional Committee for Use and Care of Laboratory Animals.

### Indirect immunofluorescence staining

Immunofluorescence staining was performed according to standard procedure^57^. Briefly, the mouse skeletal muscle cryo-sections were air dried at room temperature and fixed with cold methanol (4 °C) for 15 min. The fixed sections were permeabilized with 0.1% Triton X-100 at RT for 15 min, followed by blocking with diluted swine serum (1:20) for 1 hr, incubation with diluted primary antibodies for 1 hr and with secondary antibodies for 45 min. Nucleus was counterstained with DAPI. Images were taken by using SP5 confocal microscope (Leica, Germany), and were processed by Adobe Photoshop software. Magnification was indicated in figure legends as scale bars.

### Statistical analysis

Data were expressed as the mean ± SEM and analyzed using GraphPad Prism 5 software with t-test for pair-wise comparisons or analysis of variance (ANOVA) and significance was depicted by asterisks, *p < 0.05, **p < 0.01.

## Additional Information

**How to cite this article**: Zhao, Y. *et al*. *XBP1* splicing triggers *miR-150* transfer from smooth muscle cells to endothelial cells via extracellular vesicles. *Sci. Rep.* **6**, 28627; doi: 10.1038/srep28627 (2016).
